# Influence Mechanisms of Community Sports Parks to Enhance Social Interaction: A Bayesian Belief Network Analysis

**DOI:** 10.3390/ijerph19031466

**Published:** 2022-01-27

**Authors:** Yawen Sun, Shaohua Tan, Qixiao He, Jize Shen

**Affiliations:** 1 Faculty of Architecture & Urban Planning, Chongqing University, Chongqing 400030, China; 20171501011@cqu.edu.cn (Y.S.); 20171501012@cqu.edu.cn (J.S.); 2 Key Laboratory of Ministry of Education of Construction and New Technology in Mountainous Town, Chongqing 400030, China; 3 College of Architecture and Urban Planning, Chongqing Jiaotong University, Chongqing 400074, China; hqx623@cqjtu.edu.cn

**Keywords:** community sports parks, social interaction, physical activity, influence mechanisms

## Abstract

Urban green spaces provide multiple ecosystem services to improve human health and well-being. Cultural ecosystem services (CES) are recognized as the most important services for urban residents through the key of social interaction. Researchers commonly acknowledge the function of community sports parks to enhance social interaction. Nevertheless, existing studies generally do not pay enough attention to the influence mechanisms of community sports parks and social interaction, especially the different types of spaces in community sports parks, which could be due to the complex feature of social interaction. This paper selects three community sports parks in Chongqing as the case study, uses BBN to identify the influence mechanisms of three common types of spaces (fitness equipment space, path space, and sports court space) in community sports parks and social interaction, aiming to explore how community sports parks enhance social interaction. The results indicated that sports court space such as basketball court and badminton court enhanced social interaction best; however, the spaces farther away from the park entrances were generally less effective in enhancing interaction. All these three types of sports spaces showed the influence mechanism of “Spatial Factors-Activity Type-Social Interaction”, while differences existed in the specific spatial influencing factors. The findings highlight that based on the BBN obtained in this study, the threshold range of spatial factors could be adjusted to enhance the effect of community sports parks on social interaction.

## 1. Introduction

Urban green spaces provide multiple ecosystem services to improve human health and well-being [1]. “Ecosystem services” (ES) are the values and benefits that people derive from functioning ecosystems [2,3]. Vegetation in urban green spaces control climate, buffer noise, and filter air pollutants [4]. These ecosystem regulation services may prevent humans from diseases and promote healthy behavior through the provision of cultural ecosystem services (CES) [5,6]. CES are recognized as the most important services for urban residents which represent some of the most familiar and personal experiences of environment that people encounter in the cities [7,8,9]. Among CES, social interaction is regarded as a key to understanding the role of ecosystems in breaking down social barriers and bringing residents together [10].

Such interactions can alleviate social problems such as neighborhood conflicts and elderly isolation caused by rapid urbanization [11,12,13]. Particularly, the CES provided by the community parks that are an important place for the neighborhood to participate in activities together, meet others, and strengthen existing social ties, help enhancing social interaction and are currently receiving widespread attention [14,15]. With the increasing demand for physical exercise among residents [16], community sports parks, a new pattern of community parks, have begun to enter into the public view [16,17]. While many community green spaces include sports equipment, but they are mainly for residents’ daily leisure and relaxation with only a few basic fitness equipment inside. Unlike this type of green spaces, sport exercise is the main function of community sports park, using landscaping combined with various outdoor sports fields (including basketball court, badminton court, etc.,) in an independent land. In China, community sports park is a new type of community park, with an extended refining development.

As more and more residents tend to visit community sports parks for physical activities [17], opportunities for neighborhood contact and social interaction increase significantly, which can be achieved by the following two ways: First, physical activity mostly requires teamwork that offers good training opportunities for cultivating interpersonal communication skills [18,19]. It is a deeper and more intimate form of social interaction and can bring various socioeconomic and ethnic groups together, especially basketball and square dancing [20,21,22]. Second, physical activity have the function of upgrade self-efficacy and reduces social anxiety [18]. Previous research indicated that physical activity such as jogging and running were associated with enhancing self-efficacy [23], as well as individuals with higher self-efficacy have stronger ability to handle interpersonal relationship and their social anxiety is lower [18,24]. Meanwhile, runners seem to think of running in some way as a “social” activity [25]. Some research explore runners’ interactions with others in public places and highlight feelings of social support and inspiration generated through passing encounters with other park goers [26,27]. All these physical activities in community sports parks may make an essential contribution to enhancing social interaction, which are important to encouraging social interaction and leading a healthy lifestyle among residents.

Since their potential, a large number of natural experiments have shown that the spatial factors in community parks influence physical activity and social interactions. Moulay A. found that park space patterns with fewer visual obstacles and clear structure influenced duration of use, which in turn increased the potential for social interactions [28]. McCoemack et al. concluded that people were more likely to choose parks with public toilets, seats, and children’s playground for social interaction [29]. At the same time, the influence of non-spatial factors has also been suggested. For example, Staats and Hartighty showed that social relationship directly influenced the quality of personal interactions in public place [30]. Cox et al. suggested that young people were more likely to use parks for high-intensity social activity such as physical exercise, while older adults tended to engage in low-intensity social activity such as leisure and relaxation [31]. Duan explored the effect of age on social behavior in parks, concluding that men in parks were more active and inclined to engage in social behavior than women [32].

While these findings provide some insight into what factors influence social interaction in community sports parks (including spatial, activity, and individual differences factors), there is very limited evidence regarding the influence mechanism between them. Traditional mathematical and statistical methods such as conjoint analysis [33], structural equation modeling [14], and multiple regression modeling analysis [28], which are commonly used today, deal more with the direct and simple influence relationships. In fact, social interaction is a relatively complex process, its influence factors and the influence mechanism between these factors and social interaction may be multiple. A suitable method for model construction and meaningful interactions is a key point to clarify such complex relationships.

Graphical models, in particular, Bayesian belief network (BBN), are well-suited to deal with such complex problems because of their capability to combine the robust probabilistic methods with the lucidity of graphs that encode causal relationships between variables and [34], as such, offer a model structure for deriving uncertainty, unpredictability, imprecision, and complexity with the machine learning algorithm [35,36,37]. The BBN is also in line with the human ecological framework [38], which can deal with a large set of variables. It is a modeling tool that has already been successfully applied in the research field of environment and physical activity [39,40].

Additionally, different types of sports spaces in community sports park such as fitness equipment space, path space, and sports court space may have different influence mechanisms for social interaction. To our knowledge, not many studies addressed differences between various types of sports spaces in community sports park.

In this study, a BBN is used, therefore, is to better understand community sports park and how to enhance social interaction by considering multiple factors. The aims of this study are: (1) To clarify the influence mechanisms of enhancing social interaction with different types of sports spaces in community sports park; (2) to explore which is the most conductive type of space to enhance social interaction; (3) to find out the factors’ influence intensity of different types of sports spaces in community sports park. The results of this study will provide strategy or spatial optimization for community sports parks construction for enhancing their social interaction function in the future.

## 2. Social Interaction

Social interaction is defined as a process of reciprocal stimulation and interactivity between at least two people [41]. It can be measured through the amount of time people spend in the space, reflecting their engagement in public space and the intensity of interaction [42,43]. The space plays a role in bringing strangers from all walks of life together to become more familiar through congregate, meeting, and participating in activity together. Based on these key components of spatial social interactions, crowds congregate and engagement with the park are two important dimensions of social interactions measured in this study. Crowds congregate represents the opportunities for social contact; engagement with the park represents the intensity of interaction. Thus, variables for social interaction measurement are clear.

## 3. Methods

### 3.1. Sample Area

**Figure 1:**
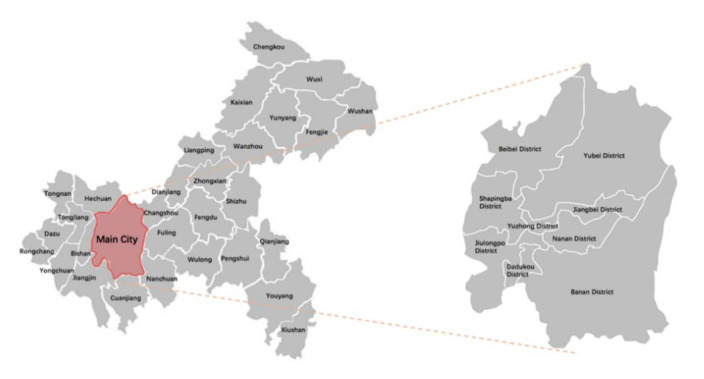
Map of Chongqing.

Chongqing is located in southwestern China and surrounded by mountains (Figure 1). Due to the topographical peculiarities, land use for community sports parks is limited. The Corner Land Project, turning “waste into treasure”, is trying to transform the scattered idle land around communities into community sports parks in different batches, solving the problems efficiently that are very popular among residents. Taking this project as a sample area may provide a good reference to understand the social interaction in community sports parks in other Chinese cities and cities in developing countries which are confronted with a similar urban context of limited land use. Finally, we selected Dashuijing, Huilongwan, and Danlong Community Sports Park (Table 1) as case study for three reasons.

First, we investigated twenty community sports parks in this project randomly in different batches and distributed them into three size categories: small, <0.5 ha; medium, 0.5–1 ha; and large, >1 ha, based on the classification of “Chongqing Community Sports and Cultural Park Construction Guidelines”. The selection aims to represent each size category.

**Figure 2:**
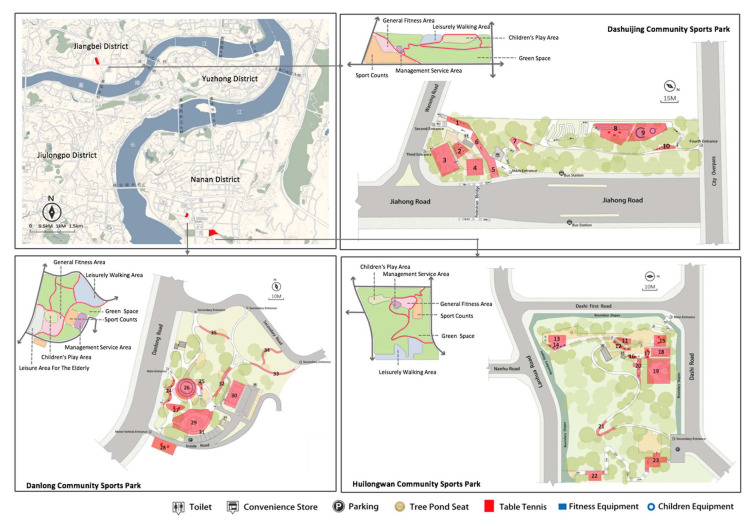
Location and layout of the three community sports parks in main city area. Rea color refers to the study units with numbers.

Second, all of them are located in old city areas of different districts (Figure 2), and insufficient sports equipment was a major problem. For example, seriously damaged and single type equipment were common. Motivation of the project offered more spaces and convenience for residents, as well as achieving a good effect of promoting physical exercise and community interaction. In addition, these three parks all belong to the “First Batch of Parks” to be opened in 2019, which are typical cases for this study.

Third, these parks construction style and inside function are similar, including fitness equipment, path, and sports court three common types of spaces that are well suited to our study needs. Based on field observation, we divided the parks into 35 spatial study units with these three different types of spaces to expand an in-depth study (Figure 2 and Table 2).

### 3.2. Data Collection

Social interaction was measured by crowds congregate and engagement with the park, in which the basic data collection of crowds congregate was mainly by on-site survey and behavior mapping. Engagement with the park was measured by questionnaire survey, and 353 valid questionnaires were collected (124 from Dashuijing, 133 from Huilongwan and 96 from Danlong). Then, the specific data acquisition methods for both are as follows.

(1) Crowds congregate

For each spatial study unit, the real distribution location of people was recorded through on-site survey, and the congregate times, total number of crowds congregate, and size of the congregate group were counted as the original data and calculated numerically by Formula (1). In order to ensure that the numerical quantification results of the trend of crowds congregate per unit area are more obvious and easier to compare and analyze, Formula (1) normalized the value of the level of crowds congregate, which can be expressed as follows.

(1) $$R=\frac{\sum_{i=1}^{N}\left(B_{i}/A_{i}\right)\times lnT_{i}}{NlgS}$$

where, *A_i_* is the average total number of crowds congregate in the space; the same spatial study unit carries different group sizes of engagement, *B_i_* is the size of the congregate group; *T_i_* is the congregate times (min); *N* is the total number of people present in the space; *S* is the area of the spatial study unit (m^2^).

Considering that the total number of crowds congregate in the space may change during the social interaction, *A_i_* is the average total number of crowds congregate in the space. So, the observation time is limited, as the time *T_i_* in formula (1). According to the collection of basic data, the interaction congregate of people in the survey space would basically occur after more than 60 min for a large-scale membership change. Therefore, the observation time (Ti) was set in the range of 1–60 min. The calculation of *A_i_* can be expressed as follows.

(2) $$A_{i}=\frac{A_{ii}+A_{io}}{2}$$

where, *A_ii_* is the total number of crowds in the space at the beginning of the interaction, *A_io_* the total number of crowds in the space at the end of the interaction, each person is assumed to have appeared in the space only once.

(2) Engagement with the park

Engagement with the park was measured by a questionnaire which consists of three parts, first part “The use of community sports parks”, second part “Engagement with the park” and third part “Personal situation” (Appendix A). The engagement quantitative results mainly came from the questionnaire’s second part that contained the following three points: (1) Subjective scoring the intensity of individual’s participation in the parks’ activity; (2) the intensity of willingness to use this space as the preferred activity space; (3) individual’s frequency of daily visits. This part of questionnaire used a scoring system, with scores from 1 to 7 indicating “low” to “high”, and the final engagement level was obtained by averaging the scores of the three questions.

However, engagement with the park would be obviously influenced by the individuals’ activity purposes. In order to ensure the accuracy of the average score results, before the questionnaire survey, we investigated the activity types in different spatial study units and summarized them (Table 3). The questionnaire survey was not random but covered all types of activity and took its proportion as reference. For example, in fitness equipment space, there were a lot of fitness equipment and chatting activities, hence we chose more people to complete the questionnaire, and chose only a few people who participated in relaxation and photo shoot activities to complete the questionnaire with not ignoring them.

The data collection was conducted on sunny days in April–May 2020, included two weekdays and two days, during the morning (7:30–11:30), afternoon (3:00–6:00), and evening (7:00–9:30). The selected spatial variables influencing community sports park’s interaction and their quantitative measures are shown in Table 4. For the non-spatial variables, activity type was measured by the presence or absence of sports activity, and leisure activity in the space was recorded on site. The specific classification and proportion of these two types of activity are shown in Table 3; individual difference included age, gender of the residents, and social relationship of their interaction partners. Social relationship reflects the range from alone, general neighborhood, familiar neighborhood, to family or friend relationship. All the variables used in further BBN are shown in Table 5.

### 3.3. Bayesian Belief Network

The BBN was used to analyze the relationship between spatial, activity, and individual difference and social interaction variables. The BBN consists of two parts: One is the directed acyclic graph (DAG), which represent the independence and causality relationships of the variables. Through machine learning algorithm, when the DAG shows a link of A → B (both A and B are variables), it represents the causality relationship. A is called a parent of B, B is a child of A (B could be the parent of another variable). Such as in this study, a link between physical activity and crowds congregate may be found, with physical activity is the parent of crowds congregate. The other is the conditional probability table (CPT), which represents the dependence level of a child variable on its parent variable. For example, “CPT for A → B” means the probabilities for each level of crowds congregate (from very low to very high) depend on the availability of physical activity (yes or no). Thus, we used the machine learning algorithm to separately modeling BBNs for each type of sport spaces in the sample community sports parks. The entire process of BBN modeling was conducted in Netica [46], and included three steps.

- First step: Draft network

In order to improve the efficiency of machine search in the subsequent stages, the network of influence links between variables was initially constructed using a priori knowledge derived from empirical data, relevant studies or expert knowledge, it was the initialization of the DAG [47].

- Second step: Training/calibration network

This step was as following: (1) Divided the sample data into a training set and a test set which were not overlapping. (2) Used the machine learning algorithm model to randomly create or remove dependencies between any two variables to generate a complete sketch of network relationships as the candidate network. (3) Adjusted the CPT to optimize the joint conditional probabilities for training each candidate network with the expectation–maximization (EM) algorithm method in the training set. The EM algorithm updated initial parameter estimates by iteratively refitting the case file data to the final model till convergence and minimizes negative log likelihood [48]. Through this step, we obtained some relatively high-quality networks.

- Third step: Test network

Finally, these candidate networks were tested. The resulting candidate networks were randomly tested for accuracy on the test set, and the network with the highest accuracy was selected as the final influence mechanisms. Because in this study, we are trying to confirm which and how the factors influence social interaction in community sports parks. So, the social interaction variable should be treat as criteria. The error rates of the crowds congregate variable and engagement with the park variable were calculated to achieve a probabilistic predictive evaluation of the network’s accuracy and precision. The calculation method is logarithmic loss, whose value is determined solely by the probability of the actual occurrence of the outcome [49], and the closer the value is to zero, the better the accuracy performance of the network is represented [50]. The final three types of sport spaces’ BBNs which were selected achieved accuracy rates of 74% crowds congregate and 76% engagement with the park in fitness equipment space, 56.25% and 75% accuracy in path space, 78% and 69% accuracy in sports court space.

We obtained the BBNs for fitness equipment space, path space, and sports court space, which represented the influence mechanisms to enhance social interaction in community sports parks. The CPT belongs to BBNs that predicted and analyzed the different level of crowds congregate and engagement with the park (as child variables) depended on its parent variables. At last, the BBNs model evaluation was necessary, it was possible to identify the most influential factors and the causal relationships of importance [50]. We applied the mutual information (entropy reduction) function in Netica for sensitivity analysis to evaluate the variables with greater influence on the target variables “crowds congregate” and “engagement with the park” [34]. Mutual information is symmetric between the two variables and is a predictor of the extent to which a finding of one variable (findings or explanatory variable) is expected to alter the beliefs (measured as entropy reduction) of another variable (query variable) [50,51,52]. The Netica software calculated the entropy reduction of other variables in the network, expressed as the query variables or as a percentage of the total entropy of the variables.

## 4. Analyses and Results

### 4.1. Social Interaction Features Analysis of the Community Sports Parks

**Figure 3:**
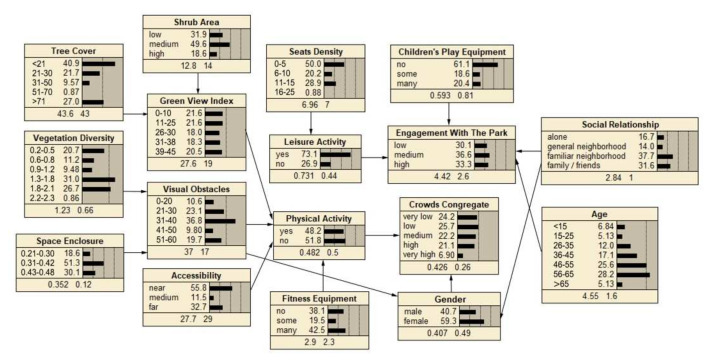
BBN modeling of fitness equipment space.

**Figure 4:**
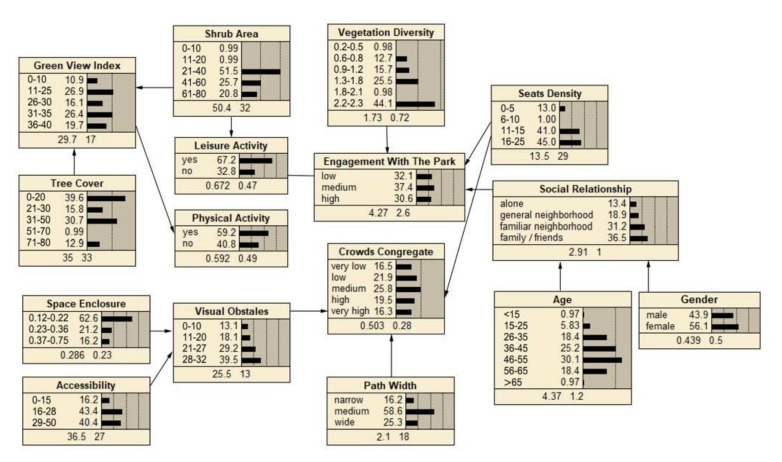
BBN modeling of path space.

**Figure 5:**
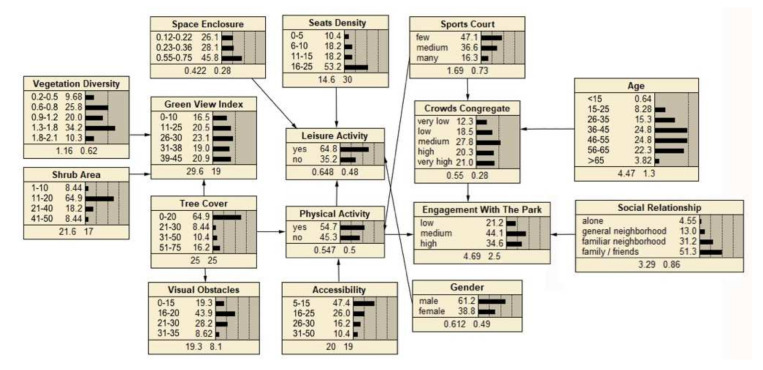
BBN modeling of sports court space.

The descriptive statistics of each node variable are shown in the network of Figure 3, Figure 4 and Figure 5, at the bottom number of each node box are indicated the means and standard deviations. The social interaction features of the three type spaces were analyzed in terms of the level of crowds congregate and engagement with the park, in which the level of crowds congregate was ranked as “sports court space (0.550) > path space (0.503) > fitness equipment space (0.426)”, and the level of engagement with the park was ranked as “ sports court space (4.69) > fitness equipment space (4.42) > path space (4.27)”. It could be concluded that the sports court space in community sports park was most conducive to promoting social interaction.

At the same time, analysis of the location of the spatial units showed that the crowds tended to congregate and interact near the park entrances, for example, the fitness equipment spaces with better congregate level were the unit of NO.1, NO.2, NO.24, and NO.26; the path spaces were the unit of NO.6, NO.7, NO.20, NO.22, and NO.32; the sports court spaces were the unit of NO.3, NO.4, NO.5, NO.17, NO.19, and NO.20, which were relatively close to the entrances rather than the inside of the park. 

### 4.2. The BBNs of Community Sports Parks Enhancing Social Interaction

#### 4.2.1. Fitness Equipment Space

The BBN of fitness equipment space to enhance social interaction is shown in Figure 3. In general, the results showed that the relationship between spatial factors and social interaction was mediated by the type of activity, with physical activity being the moderating variable for the level of crowds congregate and leisure activity for the level of engagement with the park.

Among the spatial factors, green view index, accessibility, visual obstacle and fitness equipment were all indirectly associated with the crowds congregate, which was mediated by the physical activity. Seats density was indirectly associated with engagement with the park, which was mediated by the leisure activity. Simultaneously, engagement with the park was indirectly influenced by children’s play equipment.

Another important factor that influenced social interaction was the individual difference. Gender influenced crowds congregate; age and social relationship influenced engagement with the park.

#### 4.2.2. Path Space

The BBN of path space to enhance social interaction was shown in Figure 4. In general, crowds congregate was mainly influenced by the spatial perception structural factors of visual obstacle, path width, and seats density.

However, the factors that influenced engagement with the park were relatively complex, which were moderated by both leisure activity as a mediating variable and the direct influence of vegetation diversity, seats density, and social relationship. As the unique moderating variable, leisure activity was also influenced by the shrub area.

#### 4.2.3. Sports Court Space

The BBN of sports court space to enhance social interaction was shown in Figure 5. The relationship between spatial factors and engagement with the park was mediated by sports activity, and sports activity directly influenced leisure activity; at the same time, engagement with the park was directly influenced by the crowds congregate.

In terms of spatial factors, space enclosure, accessibility, tree cover, and sports court directly influenced physical activity, while seats density directly influenced leisure activity.

In terms of individual difference factors, crowds congregate was influenced by age, and engagement with the park was influenced by social relationship.

### 4.3. Conditional Probability of Factors on Social Interaction

Table 6 and Table 7 show the conditional probabilities between all social interaction levels and their corresponding direct influence factors in the three types of spaces, which explained the BBNs in detail. In Table 6, such as path space with level of narrow (<1.5 m) path width had a slightly higher probability (19.3%) of very high level of crowds congregate than the level of medium (1.6–2 m, 17.9%) and wide (2.1–3.5 m, 18.2%) path width. Sports court space with many sport courts (more than three courts) had a slightly higher probability (21.1%) of very high level of crowds congregate than the level of medium (two courts, 19.5%) and few (only one courts, 20.8%) sport courts.

In Table 7, fitness equipment space with many children’s play equipment (more than three) had a higher probability (37.2%) of high level of engagement with the park among individuals than without (31.4%) and some (one or two, 33.9%) children’s play equipment. However, there is one exception, 36.4% of the respondents had a medium level of engagement with the park when without children’s play equipment, the children’s play features might explain this finding because they could play games and interact with friends even through a leaf or a stone, sometimes, equipment for them were absolutely unnecessary. We barely explained conditional probabilities between spatial factors and social interaction, which claimed the differences in park design lead to various uses and consequently for residents’ social interaction. Thus, compared with the non-spatial factors, adjusting the spatial factors to enhance social interaction could be more implementable.

### 4.4. Sensitivity Analysis for Factors’ Influence Intensity on Social Interaction

**Figure 6:**
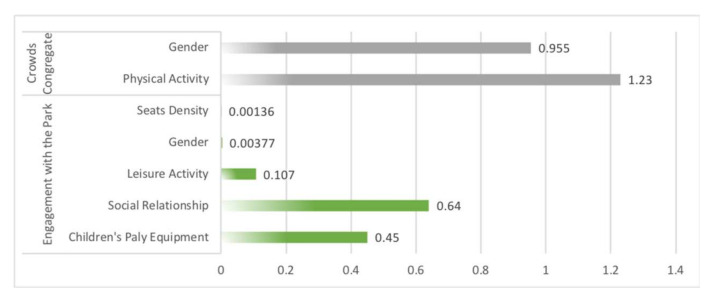
Sensitivity analysis on variables “crowds congregate” and “engagement with the park” using the entropy reduction (mutual information) of fitness equipment space.

**Figure 7:**
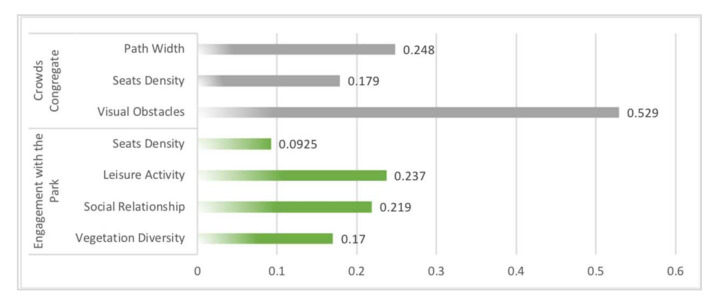
Sensitivity analysis on variables “crowds congregate” and “engagement with the park” using the entropy reduction (mutual information) of path space.

**Figure 8:**
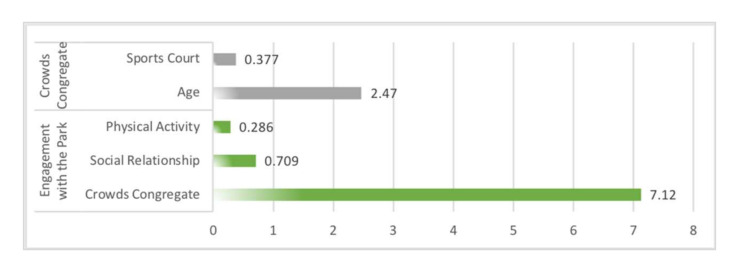
Sensitivity analysis on variables “crowds congregate” and “engagement with the park” using the entropy reduction (mutual information) of sports court space.

Sensitivity analysis in Netica (Norsys Software, Verison 6.07) obtained the strength of the relationship between the influence variables on social interaction. In fitness equipment space, the main influences on crowds congregate were physical activity (1.23) and gender (0.955), the main influences on engagement with the park were social relationship (0.64) and children’s play equipment (0.45) (Figure 6). In path space, the main influences on crowds congregate were view obstacle (0.529) and path width (0.248), the main influence on engagement with the park was leisure activity (0.237) (Figure 7). In sports court space, engagement with the park (7.12) had the greatest influence on crowds congregate, then the influence of the social relationship (0.709) was found to be significant to crowds congregate, while engagement with the park was mainly influenced by age (2.47) and sports court (0.377) (Figure 8).

## 5. Discussion

### 5.1. The Sports Court Space Is Most Conducive to Enhance Social Interaction

In this study, three types of spaces in community sports parks were identified (fitness equipment space, path space, and sports court space), and their influence mechanisms with social interaction appeared to be different. Through the comparison, we found that sports court space enhanced social interaction best, which means that in the future spatial design of community sports parks, provision of basketball courts, badminton courts, and other sports courts should be given priority. Meanwhile, attention should be paid to the environmental quality design of spaces farther away from the community sports parks’ entrances to enhance the efficiency of space use.

### 5.2. The Influence Mechanisms to Enhance Social Interaction with Three Types of Sport Spaces in Community Sprots Parks Are Different

The current study found that the influence mechanisms of three types of spaces to enhance social interaction had common features. First, engagement with the park in different types of spaces all influenced by social relationship. Second, the seats were the most important feature for encouraging leisure activity such as chatting and relaxing. Third, social interactions in three types of spaces were influenced by the mediating variable of “activity”. Additionally, the influence mechanisms also had significant differences.

#### 5.2.1. Fitness Equipment Space: Physical Activity Mediate Crowds Congregate, Leisure Activity Mediate Engagement with the Park

**Figure 9:**
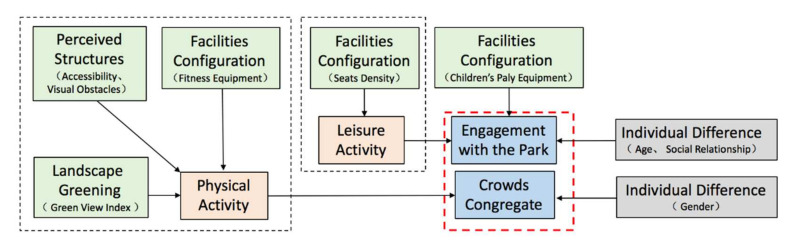
Influence mechanism of fitness equipment space to enhance social interaction.

In fitness equipment space (Figure 9), physical activity mediated crowds congregate, and the spatial factors influenced physical activity with fitness equipment, accessibility, view obstacle, and green view index, consistent with the findings of an Australian study, which showed that outdoor fitness equipment was effective in encouraging social interaction [53]. Results from our survey suggest, the usage of fitness equipment was higher, closer to the park entrance, which supported previous research indicating the relationship between accessibility and park social interaction [30]. Obstructed views influence people’s perception of space safety, which in turn influenced the activity and social interactions [28]. Aram F et al. argued that crowds always tended to socialize in places with greenery [54].

Furthermore, leisure activity mediated engagement with the park, and the spatial factors influenced leisure activity including seats density. It could be indicated that seats supported the leisure activity such as chatting, which were more likely to enhance deep social interaction. Children’s play equipment had a direct influence on engagement with the park. Previous studies on both children and adolescents’ social interaction suggested that the provision of playground/outdoor fitness equipment might be a best way to encourage park visitation [55]. 

Another important factor that directly influenced social interaction was individual difference, social relationship, and age influenced engagement with the park, gender influenced crowds congregate. For the common reason that most of the people are accompanied by friends and family when visiting the park, highlighting the importance of social relation aspects of park use [33]. Previous study showed that adults were more frequently observed using outdoor fitness equipment, with very low numbers of older adults [56,57,58]. However, our study result was in contrast to these previous studies indicated that fitness equipment might appeal to a variety of old adults. That is no surprise that elderly using parks for fitness activity is a common phenomenon in China. As more and more youngers have a stressful life and do not have enough time for outdoor exercise, which means, underscoring the importance of the park is an attractive feature for this target group [58]. Furthermore, compared with men, women interact with each other in public space usually need a higher perceived safety [59,60], which could explain the association between gender and crowds congregate in this study.

#### 5.2.2. Path Space: Leisure Activity Mediate Engagement with the Park, Spatial Factors Directly Influence Crowds Congregate

**Figure 10:**
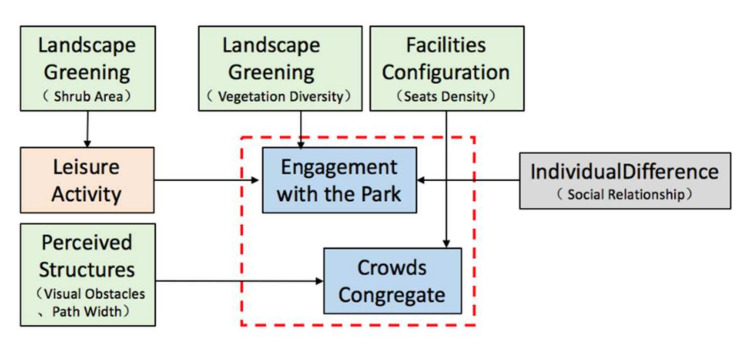
Influence mechanism of path space to enhance social interaction.

In path space (Figure 10), leisure activity mediated engagement with the park, and leisure activity was influenced by shrub area. At the same time, engagement with the park was directly influenced by vegetation diversity. Overall, the results showed that landscape and greening were the main influence factors of engagement with the park. On the one hand, individuals always tend to interact in spaces with rich landscape vegetation; on the other hand, shrubs can provide sense of refuge [61,62], which is a prerequisite key for promoting the perception of safety in individuals’ interaction.

Crowds congregate was mainly influenced by view obstacle, path width, and seats density. Visually linked will increase a sense of safety for engagement and interaction [28], and a prope path width can also enhance active interaction; the factors of view obstacle and path width influenced crowds congregate interaction through psychological perception and physical distance aspects. In addition, parks with benches are more popular due to the opportunity to alternate between physical and leisure activity and to rest after physical activity [63], which invariably increases the opportunities for interaction among strangers.

#### 5.2.3. Sports Court Space: Engagement with the Park Is Mediated by Physical Activity and Directly Influenced by Crowds Congregate

**Figure 11:**
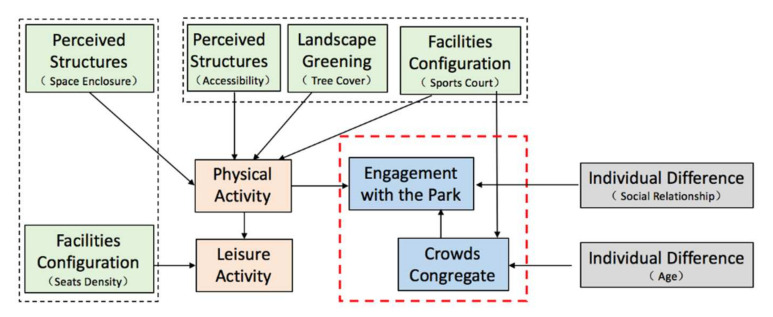
Influence mechanism of sports court space to enhance social interaction.

In sports court space (Figure 11), physical activity mediated engagement with the park, while physical activity was mainly influenced by sports court, accessibility, tree cover, and space enclosure. Previous evidence had shown that physical activity such as basketball, badminton, and square dancing had a positive impact on deep interaction [20,21,64], which was intrinsic to the influence of sports court on individuals’ park engagement. The spaces near park entrances were more preferred by users, and directly linked the relation between accessibility and physical activity. Adequate tree cover can also provide resting shade spaces, especially in hot summer, which is suitable with residents’ physical activity needs. Sports court without fences seemed to be more popular. It could attract more people and lead to a better engagement with the park activity.

Because activity features in this type of space are obvious, especially basketball and square dancing, the congregating number of people are usually above 10. These activities always require a higher and closer cooperation among individual members [19,20,21], and the participation is a deep interaction, leading to the results of crowds congregate influence individuals’ engagement with the park. Moreover, research survey showed that watch the game (13%) accounts for the most among leisure activity. More seats provided facilities for long-time game watching, therefore seats density is significantly influenced with leisure activity.

Compared with the other two types of spaces, activities in sports court space were mainly high-intensity ball sports, it would limit the participation of older adults, which was the intrinsic reason for the association between age and engagement with the park.

### 5.3. Factors’ Influence Intensity with Three Types of Sports Spaces in Community Sports Parks

In terms of the intensity of influence factors, non-spatial factors were the ones that strongly influenced all three types of spatial interactions. For example, physical activity (1.23) and gender (0.955) had the strongest influence on fitness equipment space interactions, age (2.47) also had a stronger influence on sports court space interactions. The reason may be that the entropy reduction function analysis in Netica was only able to identify the intensity of the direct influence variables on the target variables. Furthermore, the influence mechanism of “Spatial Factors-Activity Type-Social Interaction” could be constructed for all three types of spaces. Based on the BBNs obtained in this study, the threshold range of spatial factors could be adjusted to enhance the effect of community sports parks on social interaction.

### 5.4. Limitations

This study has a few limitations. The research procedure gave less insight regarding external factors of the sample study units which may influence park social interaction. First, most communities in China are closed, the other sports equipment inside the community was ignored, they may have an impact on the park use. Second, we did not consider the size of community sports parks. Previous research indicated that park size influenced individuals’ use and might provide different attributes of human social interaction and well-being [65], which is possible that there is, to some extent at least, a two-way relationship between park size and the individuals’ use of our case study. Future studies should explore this topic that shed more light on the existence and the direction of causality. 

## 6. Conclusions

This study exposes the existing problems in planning and construction of community sports parks, which may be critical for the residents’ social health benefits and enhancing parks’ efficacy of interaction. This study compared the level of enhanced social interaction with the different types of sports spaces in community sports parks. Sports court space was found to be associated with a higher level of social interaction. The findings highlight the influence mechanisms of fitness equipment space, path space, and sports court space to enhance social interaction based on the obtained BBNs. The factors’ influence intensity in each type of space was calculated which directly influenced social interaction. Based on the influence mechanisms and factors’ influence intensity, the threshold range of spatial factors could be adjusted to a certain extent, providing theoretical references for the construction and spatial design to enhance the effect of community sports parks on social interaction in the future.

## Figures and Tables

**Table 1:** Basic overview of the three community sports parks.

Park Name	Location	Site Area	Surrounding Land Use	Inside Function	NO. and Types of Sports Equipment	Green Ratio
Dashuijing Community Sports Park	Intersection of Jiahong Road and Wanxing Road, Jiangbei District	11,858 m^2^	Mainly residential	General fitness area, Children’s play area, Sport counts, Management service area, Leisurely walking area	15 fitness equipment, 3 children’s play equipment, 1 basketball court, 2 badminton court, 2 tennis tables, 1 plastic running track, 1 recreational walkway	40%
Huilongwan Community Sports Park	South of the intersection of Nanhu Road and Dashi First Road, Nanan District	8199 m^2^	Mainly residential, adjacent to the flower market, surrounded by factories	General fitness area, Children’s play area, Sport counts, Management service area, Leisurely walking area	11 fitness equipment, 4 children’s play equipment, 1 basketball court, 2 badminton courts, 6 tennis tables,1 plastic running track, 1 recreational walkway	38%
Danlong Community Sports Park	Southwest of the intersection of Danlong Road and Dashi Road, Nanan District	3803 m^2^	Mainly residential	General fitness area, Children’s play area, Sport counts, Leisure area for the elderly, Management service area, Leisurely walking area	6 fitness equipment, 1 children’s play equipment, 1 basketball court, 1 badminton court, 4 tennis table, 1 plastic running track, 1 recreational walkway	37.6%

**Table 2:** Different types of spaces’ corresponding unit numbers and photos.

	Space Type	Unit Numbers	Photo
Fitness Equipment Space	Equipment space	1, 2, 10, 12, 14, 24, 25	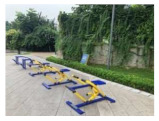
	Children’s palyground	9, 11, 26	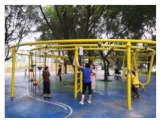
Path Space	Plastic runway	6, 20, 21, 31, 32	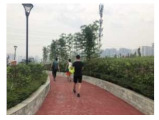
	Recreational trails	7, 22, 23, 33, 34, 35	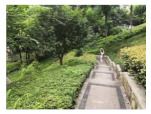
Sports Court Space	Basketball court	3, 19, 29	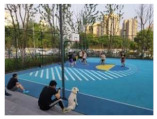
	Badminton court	4, 13, 18, 30	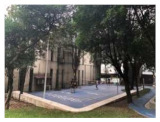
	Table tennis court	8, 15, 16, 17, 27, 28	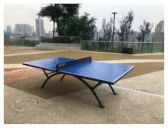
	Square dancing venues	5	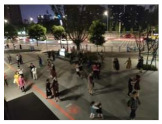

**Table 3:** Proportion of activities in three types of spaces in community sports parks.

Activities Types	Fitness Equipment Space	Path Space	Sports Court Space
Physical Activity			
Equipment Fitness	32.4%	——	——
Children’s equipment playing ^1^	18.5%	——	——
Walking	——	37.1%	——
Running	——	14.8%	——
Play basketball	——	——	14.3%
Play badminton	——	——	12.8%
Play table tennis	——	——	15.6%
Square dancing	——	——	17.5%
Leisure Activity			
Relaxation	6.3%	7.6%	11.2%
Childcare	10.5%	——	5.8%
Chatting	23.6%	12.6%	2.3%
Photo shoot	2.4%	4.5%	——
Play Mobile	6.3%	——	——
Walking the dog	——	3.2%	——
Hanging out	——	12.6%	——
Enjoy the scenery	——	3.7%	——
Family Gathering	——	2.6%	——
Neighborhood Meeting	——	1.3%	——
Watch the game ^2^	——	——	13.4%
Children playing ^3^	——	——	7.1%

—— represents no data available, and the activities are categorized by the people’s purposes. ^1^ Children’s equipment playing refers to children playing with swings, balance bars, ladders, etc. ^2^ Watch the game refers to watching the square dancing and ball games playing such as basketball, badminton and table tennis. ^3^ Children Playing refers to children play with a stone, leaf or games together, without the equipment which the spaces provide or there is no equipment provided.

**Table 4:** Spatial variables that influence the community sports park’s interaction and their quantitative measurement methods.

Variable	Variable Quantification Methods	Unit
Perceptual structure		
Accessibility	Walking distance to the nearest entrance/exit location of the park	m
Space Enclosure	Spatial enclosure = L1/L; where: L1 is the perimeter of the plan layout of trees less than 1.2 m in height and shrubs more than 1.2 m in height below the middle branch combined with the bottom part of the study plot; L is the perimeter of the study unit boundary	——
Visual Obstacles	Simulating the 125° field of view of human binocular vision [44], the camera took three photos from left to right with the spatial unit as the origin, and used AutoCAD to process the combined photos to calculate the proportion of visible space	%
Path Width	Calculated from the current site mapping	m
Facility Configuration		
Seats Density	Length of resting facilities such as benches within 100 m of each walk	m
Fitness Equipment	Calculate the number of units based on the current site mapping	——
Children’s Play Equipment	Calculate the number of units based on the current site mapping	pcs
Sports Court	Calculate the number of units based on the current site mapping	pcs
Landscape Greening		
Shrub Area	Calculated from the current site mapping	m^2^
Tree Cover	Tree cover = S1/S; where: S1 is the sum of the vertical projection of trees in the unit; S is the site area of the study unit	
Green View Index	Simulating the 125° field of view of human binocular vision, with the spatial unit as the origin, the camera took three photos from left to right and used AutoCAD to process the combined photos in order to calculate the proportion of greenery elements [45]	——
Vegetation Diversity	The Shannon–Wiener index was used to calculate diversity $H=-\overset{N}{\underset{i=1}{\sum }}P_{i}InP_{i}$, where P is the proportion of the number of vegetation species in the unit occupied by the ith number of vegetation species, i.e., $P_{i}=N_{i}/N$	——

—— represents no unit available.

**Table 5:** All the variables used in further BBN.

Variable Types	Variable Used in Further BBN
Spatial Variable	accessibility, space enclosure, visual obstacles, path width; seats density, fitness equipment, children’s play equipment, sports court, shrub area; tree cover, green view index, vegetation diversity.
Non-spatial Variable	physical activity, leisure activity; age, gender, social relationship.
Social interaction Variable	crowds congregate, engagement with the park

**Table 6:** Conditional probabilities between all levels of crowds congregate and its direct influence factors of the three types of spaces.

		Crowds Congregate				
		Very Low	Low	Medium	High	Very High
Fitness Equipment Space						
Physical Activity	yes	31.7	28.4	19.5	17.1	3.3
	no	19.3	23.6	23.9	23.9	9.3
Sex	male	31.4	27.6	19.3	17.9	3.6
	female	19.6	24.4	24.1	23.1	8.8
Path space						
Visual Obstacles	0–10	21.4	19.1	18.8	20.0	20.0
	11–20	25.1	21.8	22.3	20.0	20.0
	21–27	18.5	23.6	20.8	20.0	20.0
	28–32	17.6	18.3	19.3	20.0	20.0
Path Width	narrow	19.8	22.3	19.5	19.3	19.3
	medium	20.1	21.2	22.0	18.7	17.9
	width	19.7	22.7	20.8	18.6	18.2
Seats Density	0–5	21.4	21.0	19.5	19.1	19.1
	6–10	20.0	20.0	20.0	20.0	20.0
	11–15	18.9	20.7	21.8	19.3	18.9
	16–25	19.5	22.6	21.8	18.3	17.9
Sports Court space						
Sports Court	few	15.2	17.6	24.4	21.9	20.8
	medium	14.0	21.5	17.9	20.1	19.5
	many	17.8	22.6	20.5	17.7	21.1
Age	<15	20.0	20.0	20.0	20.0	20.0
	15–25	29.6	18.2	43.5	33.3	17.5
	26–35	12.2	29.8	26.1	15.9	15.9
	36–45	9.8	19.7	31.7	17.8	20.6
	46–55	14.0	9.0	38.5	21.3	17.3
	56–65	12.5	26.5	16.3	15.9	32.7
	>65	24.9	24.9	15.3	15.3	15.3

Unit in this table is “%”, the higher of the number, the higher probability between the two variables.

**Table 7:** Conditional probabilities between all levels of engagement with the park and its direct influence factors of the three types of spaces.

		Engagement with the Park		
		Low	Medium	High
Fitness Equipment Space				
Children’s play Equipment	no	31.4	36.4	31.4
	some	31.4	33.9	33.9
	many	31.0	31.0	37.2
Leisure Activity	yes	29.5	34.5	35.3
	no	33.3	33.3	33.3
Age	<15	31.4	33.9	34.3
	15–25	31.4	33.9	34.3
	26–35	31.4	33.9	34.3
	36–45	31.4	33.9	34.3
	46–55	31.4	33.9	34.3
	56–65	31.4	33.9	34.3
	>65	31.4	33.9	34.3
Social relationship	alone	33.3	33.3	33.3
	general neighborhood	33.3	33.3	33.3
	familiar neighborhood	33.3	33.3	33.3
	family/friends	25.9	35.9	37.6
Path space				
Leisure Activity	yes	33.3	33.3	32.3
	no	34.7	34.0	31.9
Seats Density	0–5	35.0	33.3	32.2
	6–10	33.3	33.3	33.3
	11–15	32.8	36.1	31.2
	16–25	33.1	35.6	31.3
Vegetation Diversity	0.2–0.5	33.3	33.3	33.3
	0.6–0.8	35.5	32.5	31.5
	0.9–1.2	33.5	35.5	30.5
	1.3–1.8	31.5	34.5	33.1
	1.8–2.1	33.3	33.3	33.3
	2.2–2.3	34.7	33.4	31.8
Social Relationship	alone	34.9	32.9	32.2
	general neighborhood	34.3	34.0	31.1
	familiar neighborhood	32.7	35.3	31.7
	family/friends	32.4	35.4	31.8
Sports Court space				
Physical Activity	yes	26.9	36.8	36.1
	no	26.7	42.9	30.1
Social Relationship	alone	32.8	37.8	28.8
	general neighborhood	29.1	37.6	33.2
	familiar neighborhood	22.7	36.9	40.2
	family/friends	22.0	47.0	30.3
Crowds Congregate	very low	42.6	34.1	23.2
	low	33.8	40.6	25.1
	medium	17.0	57.9	25.0
	high	18.7	34.2	47.0
	very high	21.9	32.4	45.4

Unit in this table is ”%”, the higher of the number, the higher probability between the two variables.

## Data Availability

Not applicable.

## Appendix A. The Survey Questionnaire

Hello! I am a graduate student of the School of Architecture and Urban Planning of Chongqing University. In order to enable our community sports park construction to meet your life needs to the utmost extent, and to enhance community-neighborhood communication, I would like to ask you some questions, please answer according to the actual situation. The survey results are for academic research use only, and there is no right or wrong answer. We assure not to disclose your personal information to the outside world. Therefore, please do not have any worries when filling in, and just answer according to your own real feelings. The whole process takes about 5 min, thank you for your cooperation! Please tick “√” under the option that suits your personal situation.

### Appendix A.1. The use of Community Sports Parks

#### Appendix A.1.1. The Name of the Park You Are Currently in

□ Dashuijing Community Sports Park □ Huilongwan Community Sports Park □ Danlong Community Sports Park

#### Appendix A.1.3. The Type of Activity You Are Currently Participating in

□ Children’s equipment playing, □ Fitness equipment, □ Walking, □ Running,

□ Square dancing, □ Enjoying the scenery, □ Playing basketball, □ Playing badminton, □ Playing table tennis, □ Watching games, □ Walking the dog, □ Relaxation, □ Childcare, □ Photo shoot, □ Playing mobile, □ Family gathering, □ Chatting,

□ Hanging out, □ Neighborhood Meeting, □ Children playing, □ etc.

#### Appendix A.1.4. Do You Participate in This Type of Activity Alone (If Not, Please Fill in the Next Question)?

□ Yes □ No

#### Appendix A.1.5. The Type of Social Relationship between You and Your Partner

□ No partner □ General neighbors □ Familiar neighbors □ Family/Friends

### Appendix A.2. Engagement with the Park

#### Appendix A.2.1. What Do You Think about the Extent to Which Type of Activity You Are Currently Participating in Has Promoted Social Interaction?

□ Deep low □ Very Low □ Low □ General □ High □ Very High □ Deep High

#### Appendix A.2.2. The Intensity of Your Willingness to Choose This Space Unit as Your Preferred Activity Space

□ Deep low □ Very Low □ Low □ General □ High □ Very High □ Deep High

#### Appendix A.2.3. The General Frequency of Your Daily Visit to This Park

□ Deep low □ Very Low □ Low □ General □ High □ Very High □ Deep High

### Appendix A.3. Personal Situation

#### Appendix A.3.1. Your Gender

□ Male □ Female

#### Appendix A.3.2. Your Age

□ Under 14 □ 15–25 □ 26–35 □ 36–45 □ 46–55 □ 55–65 □ Over 65

#### Appendix A.3.3. Your Education Level

□ Elementary School □ Junior High School □ Senior High School/Technical School □ Junior Technical School □ Senior Technical School □ College □ Undergraduate □ Postgraduate

#### Appendix A.3.4. Your Occupation

□ Heads of state agencies, party organizations, enterprise networks, and public institutions
□ Professional skill worker
□ Clerks and related personnel
□ Commercial and service industry personnel
□ student
□ Skilled workers
□ Unprofessional workers
□ Self-employed
□ Temporary worker
□ other

#### Appendix A.3.5. Your Financial Income

□ Below 1000 yuan □ 1000–3000 yuan □ 3000–5000 yuan
□ 5000–10,000 yuan □ 10,000 yuan or more

Thank you again for your cooperation and wish you a happy life!

Appendix A is an optional section that can contain details and supplemental to the main text—this survey questionnaire would disrupt the flow of the main text but nonetheless remain crucial to understanding and reproducing the research shown, that would make the process of our survey for engagement with the park more easy and clear to understand.

## References

[1] Fe A., Nka B. (2020). Urban green spaces for the social interaction, health and well-being of older people—An integrated view of urban ecosystem services and socio-environmental justice-ScienceDirect. Environ. Sci. Policy.

[2] Costanza R., d’Arge R., de Groot R., Farber S., Grasso M., Hannon B., Limburg K., Naeem S., O’Neil R.V., Paruelo J. (1997). The value of the world’s ecosystem services and natural capital. Nature.

[3] Millennium Ecosystem Assessment (MEA) (2005). Ecosystems and Human Well-Being.

[4] Costanza R., de Groot R.D., Braat L., Kubiszewski I., Fioramonti L., Sutton P., Farber S., Grasso M. (2017). Twenty years of ecosystem services: How far have we come and how far do we still need to go?. Ecosyst. Serv..

[5] Kabisch N., van den Bosch M., Lafortezza R. (2017). The health benefits of nature-based solutions to urbanization challenges for children and the elderly—A systematic review. Environ. Res..

[6] Markevych I., Schoierer J., Hartig T., Chudnovsky A., Hystad P., Dzhambov A.M., de Vries S., Triguero-Mas M., Brauer M., Nieuwenhuijsen M.J. (2017). Exploring pathways linking greenspace to health: Theoretical and methodological guidance. Environ. Res..

[7] Larson L.R., Keith S.J., Fernandez M., Hallo J.C., Shafer C.S., Jennings V. (2016). Ecosystem services and urban greenways: What’s the public’s perspective?. Ecosyst. Serv..

[8] Dickinson D.C., Hobbs R.J. (2017). Cultural ecosystem services: Characteristics, challenges and lessons for urban green space research. Ecosyst. Serv..

[9] Andersson E., Tengö M., McPhearson T., Kremer P. (2015). Cultural ecosystem services as a gateway for improving urban sustainability. Ecosyst. Serv..

[10] Petit-Boix A., Apul D. (2018). From Cascade to Bottom-Up Ecosystem Services Model: How Does Social Cohesion Emerge from Urban Agriculture?. Sustainability.

[11] Nicholson N.R. (2012). A Review of Social Isolation: An Important but Underassessed Condition in Older Adults. J. Prim. Prev..

[12] Han B., Cohen D., Mckenzie T.L. (2013). Quantifying the contribution of neighborhood parks to physical activity. Prev. Med..

[13] Bonsang E., Van Soest A. (2012). Satisfaction with social contacts of older Europeans. Social Indic. Res..

[14] Rasidi M.H., Jamirsah N., Said I. (2012). Urban Green Space Design Affects Urban Residents’ Social Interaction. Procedia Soc. Behav. Sci..

[15] Kaźmierczak A. (2013). The contribution of local parks to neighbourhood social ties. Landsc. Urban Plan..

[16] State Sport General Administration of China (2015). https://www.sport.gov.cn/searchweb/news.jsp.

[17] Urban Planning and Natural Resources Department in Chongqing (2021). http://ghzrzyj.cq.gov.cn/zwxx_186/mtgz/202101/t20210118_8779463.html.

[18] Gallagher M.W., Payne L.A., White K.S., Shear K.M., Woods S.W., Gorman J.M., Barlow D.H. (2013). Mechanisms of change in cognitive behavioral therapy for panic disorder: The unique effects of self-efficacy and anxiety sensitivity. Behav. Res. Ther..

[19] Baskin M.L., Dulin-Keita A., Thind H., Godsey E. (2015). Social and Cultural Environment Factors Influencing Physical Activity among African-American Adolescents. J. Adolesc. Health.

[20] Woodhouse D., Conricode D. (2017). In-ger-land, In-ger-land, In-ger-land! Exploring the impact of soccer on the sense of belonging of those seeking asylum in the UK. Int. Rev. Sociol. Sport.

[21] Stone C. (2018). Utopian community football? Sport, hope and belongingness in the lives of refugees and asylum seekers. Leis. Stud..

[22] Contiero D. (2019). Dojo and traditional martial arts: A social community for physical activity and health prevention in later age. J. Sci. Med. Sport.

[23] Luszczynska A., Mazurkiewica M., Ziegelmann J.P., Schwarzer R. (2007). Recovery self-efficacy and intention as predictors of running or jogging behavior: A cross-lagged panel analysis over a two-year period-ScienceDirect. Psychol. Sport Exerc..

[24] Ren Y., Li M. (2020). Influence of physical exercise on social anxiety of left-behind children in rural areas in China: The mediator and moderator role of perceived social support. J. Affect. Disord..

[25] Hitchings R., Latham A. (2017). How ‘social’ is recreational running? Findings from a qualitative study in London and implications for public health promotion. Health Place.

[26] Krenichyn K. (2004). Women and physical activity in an urban park: Enrichment and support through an ethic of care. J. Environ. Psychol..

[27] Krenichyn K. (2006). ‘The only place to go and be in the city’: Women talk about exercise, being outdoors, and the meanings of a large urban park. Health Place.

[28] Moulay A., Ujang N., Said I. (2016). Legibility of neighborhood parks as a predicator for enhanced social interaction towards social sustainability. Cities.

[29] McCormack G.R., Rock M., Toohey A.M., Hignell D. (2010). Characteristics of urban parks associated with park use and physical activity: A review of qualitative research. Health Place.

[30] Staats H., Hartig T. (2004). Alone or with a friend: A social context for psychological restoration and environmental preferences. J. Environ. Psychol..

[31] Cox D.T., Hudson H.L., Shanahan D.F., Fuller R., Gaston K.J. (2017). The rarity of direct experiences of nature in an urban population. Landsc. Urban Plan..

[32] Duan Y., Wagner P., Zhang R., Wulff H., Brehm W. (2018). Physical activity areas in urban parks and their use by the elderly from two cities in China and Germany. Landsc. Urban Plan..

[33] Linde V.H., Ariane G., Jelle V.C., Veitch J., De Bourdeaudhuij I., Van Dyck D., Clarys P., Van De Weghe N., Deforche B. (2018). Park characteristics preferred for adolescent park visitation and physical activity: A choice-based conjoint analysis using manipulated photographs. Landsc. Urban Plan..

[34] Dlamini W.M. (2010). A Bayesian belief network analysis of factors influencing wildfire occurrence in Swaziland. Environ. Model. Softw..

[35] Martin T.G., Kuhnert P.M., Mengersen K., Possingham H. (2005). The power of expert opinion in ecological models using Bayesian methods: Impact of grazing on birds. Ecol. Appl..

[36] McCann R.K., Marcot B.G., Ellis R. (2006). Bayesian belief networks: Applications in ecology and natural resource management. Can. J. For. Res..

[37] Tremblay J.-P., Hester A., Mcleod J., Huot J. (2004). Choice and development of decision support tools for the sustainable management of deer-forest systems. For. Ecol. Manag..

[38] Bronfenbrenner U. (1979). The Ecology of Human Development: Experiments by Nature and Design.

[39] Kemperman A., Timmermans H. (2014). Green spaces in the direct living environment and social contacts of the aging population. Landsc. Urban Plan..

[40] Kemperman A.D.A.M., Timmermans H.J.P. (2011). Children’s recreational physical activity. Leis. Sci..

[41] Hari R., Kujala M.V. (2009). Brain basis of human social interaction: From concepts to brain imaging. Physiol. Rev..

[42] Carmona M., Tiesdell S., Heath T., Oc T. (2010). Public Places—Urban Spaces: The Dimensions of Urban Design.

[43] Gehl J. (2011). Life between Buildings: Using Public Spaces.

[44] Smardon R.C., Palmer J.F., Felleman J.P. (1986). Foundations for Visual Project Analysis.

[45] Han K.T. (2017). The Effect of Nature and Physical Activity on Emotions and Attention while Engaging in Green Exercise. Urban For. Urban Green..

[46] Norsys Software Corporation Netica Verison 6.07. https://www.norsys.com/download.html.

[47] Cain J. (2001). Planning Improvements in Natural Resources Management: Guidelines for Using Bayesian Belief Networks to Support the Planning and Management of Development Programmes in the Water Sector and Beyond.

[48] Lauritzen S.L. (1995). The EM algorithm for graphical association models with missing data. Comput. Stat. Data Anal..

[49] Kalácska M., Sánchez-Azofeifa G.A., Caelli T., Rivard B., Boerlage B. (2005). Estimating leaf area index from satellite imagery using Bayesian belief networks. IEEE Trans. Geosci. Remote Sens..

[50] Colwell R.G., Dawid A.P., Speigelhalter D.J. (1993). Sequential model criticism in probabilistic expert systems. IEEE Trans. Pattern Anal. Mach. Intell..

[51] Korb K.B., Nicholson A.E. (2004). Bayesian Artificial Intelligence.

[52] Pearl J. (1998). Probabilistic Reasoning in Intelligent Systems: Networks of Plausible Inference.

[53] Pollino C.A., Woodberry O., Nicholson A., Korb K., Hart B.T. (2007). Parameterisation and evaluation of a Bayesian belief network for use in an ecological risk assessment. Environ. Model. Softw..

[54] Furber S., Pomroy H., Grego S., Tavener-Smith K. (2014). People’s experiences of using outdoor gym equipment in parks. Health Promot. J. Aust..

[55] Aram F., Solgi E., Holden G. (2019). The role of green spaces in increasing social interactions in neighborhoods with periodic markets. Habitat Int..

[56] Cohen D.A., Marsh T., Williamson S., Golinelli D., McKenzie T.L. (2012). Impact and cost-effectiveness of family Fitness Zones: A natural experiment in urban public parks. Health Place.

[57] Cranney L., Phongsavan P., Kariuki M., Stride V., Scott A., Hua M. (2016). Impact of an outdoor gym on park users’ physical activity: A natural experiment. Health Place.

[58] Veitch J., Salmon J., Abbott G., Timpiero A., Sahlqvist S. (2021). Understanding the impact of the installation of outdoor fitness equipment and a multi-sports court on park visitation and park-based physical activity: A natural experiment. Health Place.

[59] Veitch J., Salmon J., Ball K. (2007). Children’s perceptions of the use of public open spaces for active free-play. Child. Geogr..

[60] Lloyd K., Burden J., Kieva J. (2008). Young girls and urban parks: Planning for transition through adolescence. J. Park Recreat. Adm..

[61] Grahn P., Stigsdotter U.K. (2013). Workplace greenery and perceived level of stress: Benefits of access to a green outdoor environment at the workplace. Landsc. Urban Plan..

[62] Stigsdotter U.K., Corazon S.S., Sidenius U., Refshauge A.D., Patrik G. (2017). Forest design for mental health promotion-using perceived sensory dimensions to elicit restorative responses. Landsc. Urban Plan..

[63] Van Hecke L., Loyen A., Verloigne M., van der Ploeg H.P., Lakerveld J., Brug J., De Bourdeaudhuij I., Ekelund U., Donnelly A., Hendriksen I. (2016). Variation in population levels of physical activity in European children and adolescents according to cross-European studies: A systematic literature review within DEDIPAC. Int. J. Behav. Nutr. Phys. Act..

[64] King K.A., Vidourek R.A., English L., Merianos A.L. (2013). Vigorous physical activity among college students: Using the health belief model to assess involvement and social support. Arch. Exerc. Health Dis..

[65] Ayala-Azcárraga C., Diaz D., Zambrano L. (2019). Characteristics of urban parks and their relation to user well-being. Landsc. Urban Plan..

